# Targeting Autophagy Triggers Apoptosis and Complements the Action of Venetoclax in Chronic Lymphocytic Leukemia Cells

**DOI:** 10.3390/cancers13184557

**Published:** 2021-09-10

**Authors:** Damjan Avsec, Alma Tana Jakoš Djordjevič, Maša Kandušer, Helena Podgornik, Matevž Škerget, Irena Mlinarič-Raščan

**Affiliations:** 1 University of Ljubljana, Faculty of Pharmacy, SI-1000 Ljubljana, Slovenia; damjan.avsec@ffa.uni-lj.si (D.A.); tanaalma@gmail.com (A.T.J.D.); masa.kanduser@ffa.uni-lj.si (M.K.); helena.podgornik@kclj.si (H.P.); 2 University Medical Centre Ljubljana, Department of Haematology, SI-1000 Ljubljana, Slovenia; matevz.skerget@kclj.si; 3 University of Ljubljana, Faculty of Medicine, SI-1000 Ljubljana, Slovenia

**Keywords:** chronic lymphocytic leukemia, autophagy, AMPK/ULK1, venetoclax, drug resistance, targeted therapy

## Abstract

**Simple Summary:**

Venetoclax is an antagonist of the antiapoptotic protein Bcl-2, and is currently approved for treatment of chronic lymphocytic leukemia (CLL). Recently, clinical use has shown that patients develop resistance to venetoclax. Therefore, the demand for novel targets for treatment of CLL remains high. One such target is autophagy, an evolutionarily old system for degradation of long-lived proteins and organelles that recovers the energy for normal cellular functions. Here, the antileukemic potential of different autophagy inhibitors was evaluated in patient-derived CLL cells. Among these, inhibitors of the AMPK/ULK1 pathway and late-stage autophagy were the most potent, with selective cytotoxic activities seen. They also show activity against CLL cells with unfavorable genetic characteristics. These inhibitors complement the cytotoxic action of venetoclax. In conclusion, targeting autophagy shows potential as a novel approach for treatment of patients with CLL.

**Abstract:**

Continuous treatment of patients with chronic lymphocytic leukemia (CLL) with venetoclax, an antagonist of the anti-apoptotic protein Bcl-2, can result in resistance, which highlights the need for novel targets to trigger cell death in CLL. Venetoclax also induces autophagy by perturbing the Bcl-2/Beclin-1 complex, so autophagy might represent a target in CLL. Diverse autophagy inhibitors were assessed for cytotoxic activities against patient-derived CLL cells. The AMPK inhibitor dorsomorphin, the ULK1/2 inhibitor MRT68921, and the autophagosome–lysosome fusion inhibitor chloroquine demonstrated concentration-dependent and time-dependent cytotoxicity against CLL cells, even in those from hard-to-treat patients who carried del(11q) and del(17p). Dorsomorphin and MRT68921 but not chloroquine triggered caspase-dependent cell death. According to the metabolic activities of CLL cells and PBMCs following treatments with 10 µM dorsomorphin (13% vs. 84%), 10 µM MRT68921 (7% vs. 78%), and 25 µM chloroquine (41% vs. 107%), these autophagy inhibitors are selective toward CLL cells. In these CLL cells, venetoclax induced autophagy, and addition of dorsomorphin, MRT68921, or chloroquine showed potent synergistic cytotoxicities. Additionally, MRT68921 alone induced G2 arrest, but when combined with venetoclax, it triggered caspase-dependent cytotoxicity. These data provide the rationale to target autophagy and for autophagy inhibitors as potential treatments for patients with CLL.

## 1. Introduction

Over the last decade, the treatment of patients with chronic lymphocytic leukemia (CLL) has undergone a radical change that has led to the advent of targeted therapies. Among the novel targeted therapies, venetoclax is a first-in-class antagonist of the antiapoptotic protein B-cell lymphoma 2 (Bcl-2). Venetoclax successfully progressed from bench to bedside [1], initially as a second-line therapy, and is now increasingly used in front-line settings for treatment-naïve patients [2]. However, the occurrence of resistance to venetoclax within 2–3 years of its initiation, which can occur even after robust and deep clinical responses [3], represents a persistent and complex problem on the path to better clinical outcomes for patients with CLL. One of the potential therapeutic targets that can be exploited for treatment of venetoclax-resistant CLL is autophagy, an evolutionarily old, degradative process responsible for the clearance of misfolded and aggregated proteins, damaged organelles, and intracellular pathogens, that provides energy and building blocks for cellular homeostasis [4,5].

The implications for targeting autophagy in cancer treatments remain controversial. This is due to both the cell-type-dependent and context-dependent functions, and their duality in cellular homeostasis, as autophagy is both a prosurvival pathway and a form of programmed cell death [5,6,7,8]. A mere handful of studies have touched on the role of autophagy in CLL, both from the prognostic point of view and for possible implications in therapy. The latter is of particular interest, as initial autophagy signaling pathways are well intertwined with the BCR signaling pathway. One such crossroad involves the PI3K/Akt/mTOR pathway, which is known to be crucial for the survival, growth, proliferation, and differentiation of B cells [9]. Asnaghi et al. showed that mTOR can serve as a regulator of cell survival and cell death signals via phosphorylation of Bcl-2 [10]. In line with this, targeting Bcl-2 and mTOR concurrently (e.g., with everolimus, temsirolimus) was shown to synergize against Bcl-2-antagonist-resistant B-cell lines and primary acute lymphoblastic leukemia cells by down-regulation of Mcl-1 [11]. Another study reported that the mTOR inhibitor CC-115 can reverse CD40-mediated resistance of CLL cells to venetoclax, through blocking CD40-induced Mcl-1 and Bcl-xL up-regulation, thus suggesting a role for mTOR in the control of the Bcl-2 family of proteins [12]. The BTK/Akt/mTOR pathway is another crossroads between the BCR pathway and autophagy. It has been shown that inhibition of BCR-mediated autophagy using the Vsp34 inhibitor VPS34-IN1 augmented the action of venetoclax in CLL [13]. Contrary to mTOR, the AMPK pathway has been less investigated in CLL; however, it has recently been implicated in energy reprogramming of venetoclax-resistant lymphoma cells [14]. AMPK can also activate the JNK stress kinase, to thus phosphorylate Bcl-2 [15], which promotes increased stability [16,17,18] and affinity of Bcl-2 for proapoptotic members of the Bcl-2 family [19]. Importantly, Bcl-2 binds to Beclin-1 to prevent the formation of Beclin-1/hVPS34 PI3K and thus autophagy [20], while retaining its full antiapoptotic function [21]. Therefore, the Bcl-2/Beclin-1 complex physically and functionally links autophagy and apoptosis, which thus proposes that Bcl-2 antagonists (such as venetoclax) can affect both systems at once.

In the present study, we investigated autophagy as a potential target for triggering cell death in CLL cells. By applying a pharmacological approach, different targets within the autophagic pathway were evaluated in patient-derived CLL cells. We further investigated the mechanism of action of autophagy inhibitors, and examined the effects of targeting autophagy in combination with venetoclax in terms of the impact on CLL cell survival.

## 2. Materials and Methods

### 2.1. Compounds

Dorsomorphin dihydrochloride (HY-13418), MRT68921 dihydrochloride (HY-100006A), MHY1485 (HY-B0795), Torin-1 (HY-13003), venetoclax (HY-15531), and quinoline-Val-Asp-difluorophenoxymethylketone (Q-VD-OPh; HY-12305) were from MedChemExpress (Monmouth Junction, NJ, USA). Chloroquine phosphate (14194), wortmannin (10010591), bafilomycin A1 (11038), rapamycin (13346), 3,3’-dihexyloxacarbocyanine (iodide) (DiOC6; 23124), and 4′,6-diamidino-2-phenylindole (DAPI; 14285) were from Cayman Chemical (Ann Arbor, MI, USA).

### 2.2. Cell Culture

MEC-1 cells were from Deutsche Sammlung von Mikroorganismen und Zellkulturen GmbH (Braunschweig, Germany) and were maintained in an Iscove’s Modified Dulbecco’s Medium (Gibco, Grand Island, NY, USA) supplemented with 10% heat-inactivated fetal bovine serum, 100 U/mL penicillin and 100 µg/mL streptomycin. THP1-Difluo hLC3 cells were from InvivoGen (San Diego, CA, USA) and were maintained in RPMI1640 medium, supplemented with 10% heat-inactivated fetal bovine serum, 2 mM L-glutamine, 100 U/mL penicillin, 100 µg/mL streptomycin, and 100 µg/mL Normocin (InvivoGen, San Diego, CA, USA). Selection of clones expressing the red fluorescent protein (RFP): green fluorescent protein (GFP): LC3 fusion protein was carried out using 100 µg/mL Zeocin (InvivoGen; San Diego, CA, USA). The cells were passaged every 2–3 days and maintained at 0.5–1.5 × 10^6^ cells/mL. Prior to experiments, the cells were washed with phosphate-buffered saline (PBS) and resuspended in Normocin-free culture medium.

Cell lines were tested for mycoplasma contamination using MycoAlert PLUS mycoplasma detection kit (Lonza Group Ltd., Basel, Switzerland) and/or Mycoplasmacheck qPCR test (Eurofins Genomics, Ebersberg, Germany).

### 2.3. Patient’s Samples

This study was approved by the Slovenian National Medical Ethics Committee (93/12/10; 0120-136/2019/4). The patients with CLL provided their written informed consent. Peripheral blood samples were collected from 28 patients with CLL (20 male; 8 female), with the median age at sample collection of 66 years (range 38–87 years) and the median time from diagnosis of 6.5 years (range 0–16 years). The diagnosis was made according to the current guidelines, with 27 patients diagnosed with CLL, and one patient with atypical chronic lymphocytic leukemia/mantle cell lymphoma. Eleven patients were treatment-naïve, and 17 patients were treated, including with novel targeted therapies. CLL samples had prognostically good as well as poor genetic lesions: 16/24 had del(13q), 3/24 had trisomy 12, 4/24 had del(11q), 3/24 had del(17p), and 5/17 had mutated immunoglobulin heavy-chain variable region (IGHV) gene (Table 1). The patient CLL cells were isolated from whole blood using the RosetteSep Human B Cell Enrichment Cocktail (Stem Cell Technologies, Vancouver, BC, Canada), as described previously [22]. Peripheral blood mononuclear cells (PBMCs) of healthy donors were also isolated as described previously [22]. Both primary CLL cells and PBMCs were maintained at 1–2 × 10^6^ cells/mL in RPMI1640 medium, supplemented with 10% heat-inactivated fetal bovine serum, 2 mM L-glutamine, 100 U/mL penicillin, and 100 µg/mL streptomycin. 

### 2.4. PrestoBlue Assay

Cell lines (3 × 10^5^ cells/mL), primary CLL cells and PBMCs (1 × 10^6^ cells/mL) were left untreated (control) or were treated with 0.2% DMSO (vehicle control) or with selected compounds. Then, 100 µL samples were added in duplicates into each well of black 96-well plates, which were incubated for 24 h or 48 h at 37 °C, in a 5% CO_2_ humidified atmosphere. Following addition of 10 µL PrestoBlue reagent (Invitrogen, Carlsbad, CA, USA) to each well, the samples were incubated at 37 °C for 1–3 h. Subsequently, the fluorescence was measured at 590 nm in a microplate reader (Synergy HTX Multi-Mode Microplate Reader; BioTek Instruments, Inc., Winooski, VT, USA). The relative metabolic activities were calculated as (I_sample_ − I_blank_)/(I_control_ − I_blank_) × 100 (%). The data are presented as means ± SEM of ≥3 independent experiments, each carried out in duplicate.

### 2.5. Imaging Flow Cytometry

#### 2.5.1. Autophagic Flux

Autophagic flux was determined using the THP1-Difluo hLC3 autophagy reporter cell line, which expresses the fusion protein RFP–GFP-microtubule-associated protein 1 light chain 3 beta (LC3B). This LC3B is incorporated into autophagic vesicles. Upon activation of autophagy, acidic autolysosomes are formed, in which RFP is stable, while GFP degrades. In contrast, when autophagy is inhibited, GFP accumulates, and RFP remains intact. The ratio of RFP to GFP fluorescence thus defines the measure of autophagic flux in cells. Cells (5 × 10^5^ cells/mL) were treated with 1 µM to 25 µM venetoclax for 24 h. The cells were then pelleted, resuspended in 50 µL PBS, and analyzed using an imaging flow cytometer (Amnis ImageStream X Mk II; Luminex Corporation, Austin, TX, USA). A minimum of 10,000 events in focus was collected per sample. The median fluorescence intensity (MFI) of RFP and GFP was assessed.

#### 2.5.2. TFEB Nuclear Translocation Assay

MEC-1 cells (1 × 10^6^) were incubated with indicated compounds or cultured in Hank’s Balanced Salt Solution (HBSS; Gibco, Grand Island, NY, USA) for 1 h. The cells were then centrifuged (200× *g*, 5 min) and fixed with 0.5 mL 4% paraformaldehyde for 10 min. There followed a wash with 0.5 mL PBS with centrifugation as before, and permeabilization with 0.5% Triton X-100 in 5% bovine serum albumin (BSA)/PBS for 20 min. Subsequently, cells were washed and incubated with 100 µL 5% BSA/PBS for 30 min. To this, 1 µL Anti-TFEB Antibody (C-6) (1:100 dilution; sc-166736; Santa Cruz Biotechnology, Dallas, TX, USA) was added and incubated at room temperature for 1 h. The cells were washed again and resuspended in 100 µL 5% BSA/PBS, to which 0.2 µL goat anti-mouse IgG-PE antibody (1:1000 dilution; sc-3738; Santa Cruz Biotechnology, Dallas, TX, USA) was added and incubated in the dark for 1 h. Then, 100 µL 6 µM DAPI was added (final concentration, 3 µM), incubated for 5 min, then the cells were washed with 0.5 mL PBS. The pellet was resuspended in 20 µL PBS and a minimum of 5,000 events in focus was collected using an imaging flow cytometer (Amnis ImageStream X Mk II; Luminex Corporation, Austin, TX, USA). The basal level of nuclear translocation of TFEB in control cells was determined by comparison of the DAPI and TFEB images using the IDEAS wizard Nuclear localization. Gating on control cells with translocated TFEB was used as the template for batch analysis of the other samples. The data are presented as mean proportions of cells with TFEB translocated to the nucleus ± SEM, from ≥3 independent experiments.

#### 2.5.3. LysoTracker/DAPI Assay

Lysosomal integrity and the viability of cells was assessed using LysoTracker Red DND (Invitrogen, USA) and DAPI (Cayman Chemical, Ann Arbor, MI, USA), respectively. The CLL cells (1 × 10^6^ cells/mL) were treated with indicated compounds for 24 h, then the culture medium was removed (centrifugation, 200× *g*, 5 min), and the cells were resuspended in 500 µL 100 nM LysoTracker Red DND-99. The cells were loaded with the probe for 1 h at 37 °C, then 3 µM DAPI was added for 10 min. The cells were then pelleted, resuspended in 20 µL culture medium, and analyzed using an imaging flow cytometer (Amnis^®^ ImageStream^®^X Mk II; Luminex Corporation, Austin, TX, USA). A minimum of 10,000 events in focus was collected per sample. Data are presented as means ± SEM of 3 independent experiments.

### 2.6. Flow Cytometry

#### 2.6.1. Cytotoxicity Assay

For determination of cell viability, the cells (1 × 10^6^ cells/mL) were left untreated (control) or were treated with 0.2% DMSO (vehicle control) or with the selected compounds. Afterward, 100 µL cell suspension was added in duplicates into each well of 96-well plates, which were then incubated for 24 h (37 °C, 5% CO_2_, humidified atmosphere). Following this incubation, propidium iodide (Molecular Probes, Eugene, OR, USA) was added to each well (final concentration, 5 µM) with 5-min incubation with gentle shaking of the plate. Then, a minimum of 10,000 events was collected using an autosampler connected to a flow cytometer (Attune NxT; Invitrogen, Carlsbad, CA, USA). The data are presented as means ± SEM of 3 independent experiments, each carried out in duplicate.

#### 2.6.2. Carboxyfluorescein Succinimidyl Ester Assay

Cell proliferation was monitored using cell proliferation kit (CellTrace CFSE; Invitrogen, Carlsbad, CA, USA), following the manufacturer instructions. Briefly, the cells were prepared in PBS at 1 × 10^6^ cells/mL and stained with 1 µM carboxyfluorescein succinimidyl ester (CFSE) for 20 min at 37 °C (protected from light). Afterward, the cells were pelleted, resuspended in culture medium, and incubated for 20 min for the final modification of the probe. Subsequently, the cells (3 × 10^5^ cells/mL) were treated with the compounds of interest for the indicated periods. Cell proliferation was then determined using a flow cytometer (Attune Nxt). A minimum of 10,000 events was collected per sample.

#### 2.6.3. Cell Cycle Analysis

The cell cycle analysis was performed in a step by step approach as described previously [22]. The proportions of the cells in each phase of the cell cycle were determined using Watson’s pragmatic model.

#### 2.6.4. DiOC6/SYTOX Red Assay

The CLL cells (1 × 10^6^ cells/mL) were treated with the compounds of interest, and then 100 µL of cell suspension was added to each well of a U-bottom 96-well plate and incubated for 24 h. Afterward, the cells were centrifuged (200× *g*, 2 min) and resuspended in 100 µL 100 nM DiOC6 (in PBS). The cells were incubated for 15 min at 37 °C (protected from light), and were then centrifuged (200× *g*, 2 min) and washed twice with 200 µL PBS. Following the washing, samples were incubated for 15 min with 100 µL 2 nM SYTOX Red Dead Cell Stain (Invitrogen, Carlsbad, CA, USA) prepared in PBS (protected from light). Finally, a minimum of 10,000 events was collected per well using an autosampler connected to a flow cytometer (Attune NxT; Invitrogen, Carlsbad, CA, USA). The SYTOX Red-/DiOC6+ indicates viable cells with high mitochondrial membrane potential, SYTOX Red-/DiOC6− indicates viable cells with compromised mitochondria, and SYTOX Red+/DiOC6− indicates dead cells. The data are presented as means ± SEM of 3 independent experiments, each carried out in duplicate.

#### 2.6.5. Annexin V/SYTOX Blue Assay

Apoptosis of primary CLL cells was assessed by determination of the levels of phosphatidylserine exposure using R-phycoerythrin conjugated annexin V (R-PE Annexin V; Invitrogen, Carlsbad, CA, USA) and the nucleic acid stain SYTOX Blue Dead Cell Stain (Invitrogen, Carlsbad, CA, USA), following manufacturer instructions, and as described previously [22]. Briefly, patient-derived CLL cells (1 × 10^6^ cells/mL) were treated with the indicated compounds for 24 h. Then, the cells were harvested, washed with cold PBS, and resuspended in the annexin-binding buffer to a final concentration of 1 × 10^6^ cells/mL. For each 100 µL of sample, 2.5 µL R-PE Annexin V was added, with incubation in the dark for 15 min. Then, 200 µL annexin-binding buffer was added, and the samples were incubated with SYTOX Blue (SB; final concentration, 750 nM) for 5 min. A minimum of 10,000 events was collected with a flow cytometer (Attune NxT; Invitrogen, Carlsbad, CA, USA). Annexin V (ANV)-/SYTOX Blue (SB)- indicates viable cells that are not undergoing apoptosis, ANV+/SB− indicates early apoptotic or proapoptotic cells, ANV+/SB+ indicates dead cells, and ANV−/SB+ indicates necrotic cells. The data are presented as means ± SEM of ≥3 independent experiments.

### 2.7. Immunoblotting

SDS-PAGE and Western blotting were carried out as described previously [22]. The anti-p62/SQSTM1 (#CY-88588) antibody was from Cell Signaling Technology (Danvers, MA, USA). The anti-β-actin antibody was from Sigma-Aldrich (1:5000 dilution; St. Louis, MO, USA). The membranes were incubated with the primary antibody (1:1000 dilution; 5% BSA/TTBS) at 4 °C overnight. After washing, membranes were incubated with the appropriate secondary antibodies conjugated with horseradish peroxidase, for 1 h at room temperature. Secondary antibodies Horse Anti-mouse IgG, HRP-linked Antibody (#7076; Cell Signaling Technology, Danvers, MA, USA) or Goat Anti-Rabbit IgG Antibody, HRP-conjugate (12-348; EMD Millipore, Burlington, MA, USA) were prepared as 1:10,000 dilution in 5% BSA/TTBS. Then, the SuperSignal West Femto substrate (Thermo Fisher Scientific, Pierce, IL, USA) was added, following the manufacturer instructions, and the chemiluminescence was measured.

### 2.8. Statistical Analysis

Data obtained with the flow cytometer were analyzed using the FlowJo_V.10.6.1 software. Data obtained with the imaging flow cytometer were analyzed using Amnis IDEAS 6.2 image analysis software. Statistical analysis was performed using the GraphPad Prism 9 software. One-way or two-way ANOVA and Student’s *t*-test were used as appropriate. Statistical significance was defined according to *p* < 0.05. ∗, ∗∗, ∗∗∗, and ∗∗∗∗ denote *p* < 0.05, *p* < 0.01, *p* < 0.001, and *p* < 0.0001, respectively.

## 3. Results

### 3.1. Autophagy Inhibitors Exert Concentration-Dependent and Time-Dependent Cytotoxicity toward Patient-Derived CLL Cells

**Figure 1:**
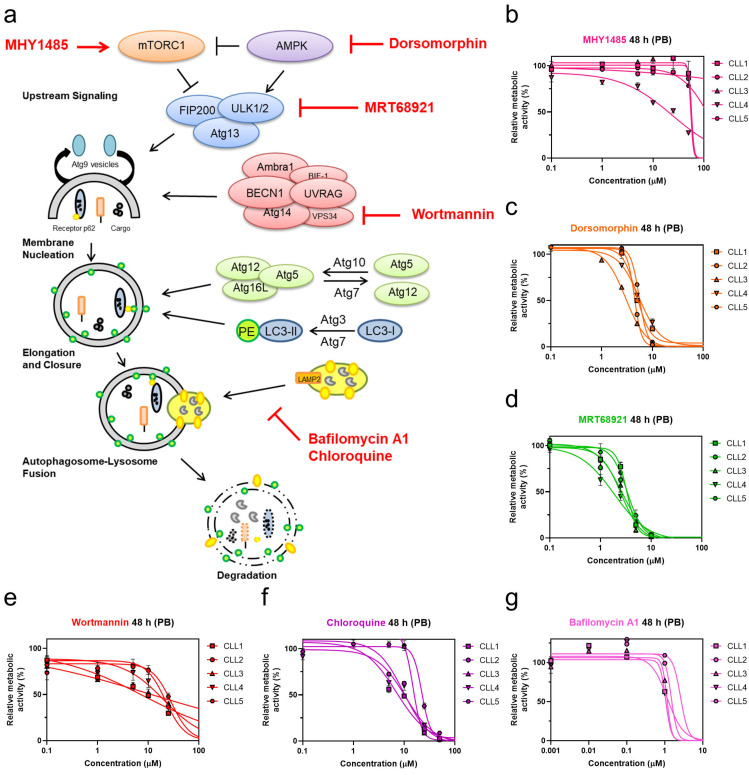
Targeting autophagy with distinct autophagy inhibitors induces concentration-dependent cytotoxicity in patient-derived CLL cells. (**a**) Overview of targets in the autophagic process and their pharmacological modulators; Determination of EC_50_ values of autophagy inhibitors (**b**) mTOR activator MHY1485, (**c**) AMPK inhibitor dorsomorphin, (**d**) ULK1/2 inhibitor MRT68921, (**e**) PI3K class III inhibitor wortmannin, (**f**) autophagosome-lysosome fusion inhibitor chloroquine, and (**g**) late-stage autophagy inhibitor bafilomycin A1 in CLL cells derived from five patients after 48 h of treatment using the PrestoBlue (PB) assay.

We initially investigated targets within the autophagic process to pinpoint the molecular switches that render CLL cells prone to undergo cell death. For this purpose, CLL cells derived from five different patients with CLL were treated with six pharmacological modulators. Prior to this, these compounds were initially shown to inhibit autophagic activity through the use of the THP1-Difluo hLC3 autophagy reporter cell line (Figure S1). After 48 h treatments of the patient-derived CLL cells with these compounds, the metabolic activities were determined using the PrestoBlue assay (Figure 1b–g).

Of the tested compounds, the most potent concentration-dependent cytotoxicity against patient-derived cells was observed for the AMPK inhibitor dorsomorphin, the ULK1/2 inhibitor MRT68921, and the autophagosome-lysosome fusion inhibitor chloroquine (Figure 1c,d,f). While the mTOR activator MHY1485 showed poor cytotoxicity at concentrations up to 100 µM (Figure 1b), the PI3K class III inhibitor wortmannin demonstrated similar cytotoxicity compared to chloroquine (Figure 1e,f) [23].

Of interest, late-stage autophagy inhibitors, chloroquine and bafilomycin A1, which both act by perturbing autophagosome-lysosome fusion, exerted different effects on CLL cells. Chloroquine was cytotoxic against primary CLL cells (Figure 1f), while bafilomycin did not affect the metabolic activity of CLL cells at concentrations used to inhibit autophagy (Figure 1g, Figure S1). As primary CLL cells do not proliferate in vitro, we tested the antiproliferative effects of compounds on CLL cell line MEC-1 [24]. Treatment of CFSE-labeled MEC-1 cells with selected compounds for 72 h revealed that only bafilomycin A1 inhibited the proliferation of cells (Figure S2).

Based on the promising antileukemic activity, the AMPK inhibitor dorsomorphin, the ULK1/2 inhibitor MRT68921, and the autophagosome-lysosome fusion inhibitor chloroquine were further evaluated for their toxicities against the full set of primary CLL cells derived from 28 patients (Table 1 and Table 2). Their mean EC_50_ values after 24 h of treatment were 6.4 µM, 4.3 µM, and 34.5 µM, respectively, which decreased further after 48 h of treatment, to 4.6 µM, 3.4 µM, and 17.8 µM, respectively. This data thus demonstrated that these three autophagy inhibitors show time-dependent cytotoxicity. MRT68921 was the most cytotoxic of these three autophagy inhibitors, followed by dorsomorphin and chloroquine. As also seen from Table 2, the ranges of EC_50_ values across the different patient-derived CLL cells was relatively wide, which indicated that in the CLL cells of some of the patients, autophagy inhibitors showed greater cytotoxicities than in others (Table 2). The EC_50_ values after 48 h of treatment ranged from 1.6 µM to 9.2 µM for dorsomorphin, from 0.2 µM to 5 µM for MRT68921, and from 2.1 µM to 24.9 µM for chloroquine. This can be attributed to CLL being a very heterogeneous disease.

**Figure 2:**
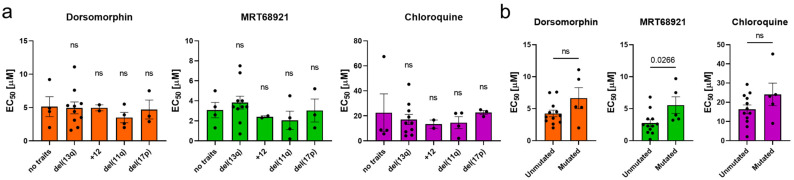
The potency of autophagy inhibitors in cells of CLL patients with different genetic characteristics. The EC_50_ values of autophagy inhibitors after 48 h with respect to (**a**) del(13q), trisomy 12 (+12), del(11q), del(17p), and (**b**) mutational status of IGHV. Data are means ± SEM. Each dot corresponds to the cells derived from a specific patient with CLL. One-way ANOVA with Dunnett’s multiple comparisons test and unpaired Student’s *t*-test were used, respectively. Not significant (ns) denotes *p* > 0.05.

To investigate whether the presence of unfavorable genetic characteristics affects the activity of autophagy inhibitors, we grouped the EC_50_ values according to genetic characteristics (del(17p), del(13q), +12, del(11q), IGHV mutational status) (Figure 2). We showed that all three autophagy inhibitors acted against cells carrying del(11q) or del(17p), thus evidencing that inhibition of autophagy is as effective in CLL cells with unfavorable genetic characteristics as in cells with favorable genetic characteristics with the exception of MRT68921, which turned out to be more effective in cells with unmutated IGHV (*p* = 0.0266) (Figure 2b).

### 3.2. Inhibition of Caspases Rescues CLL Cells from Dorsomorphin- and MRT68921- but Not Chloroquine-Induced Cell Death

The activation of caspases is one of the hallmarks of apoptotic cell death, and thus, reduced cytotoxicity of these compounds can be expected upon inhibition of caspases with the pan-caspase inhibitor (QVD-OPh) to confirm apoptosis as the mode of cell death. These patient-derived CLL cells were treated with dorsomorphin, MRT68921, and chloroquine alone or in combination with QVD-OPh for 24 h. Afterward, the cell viability was determined using propidium iodide staining and flow cytometry. 

**Figure 3:**
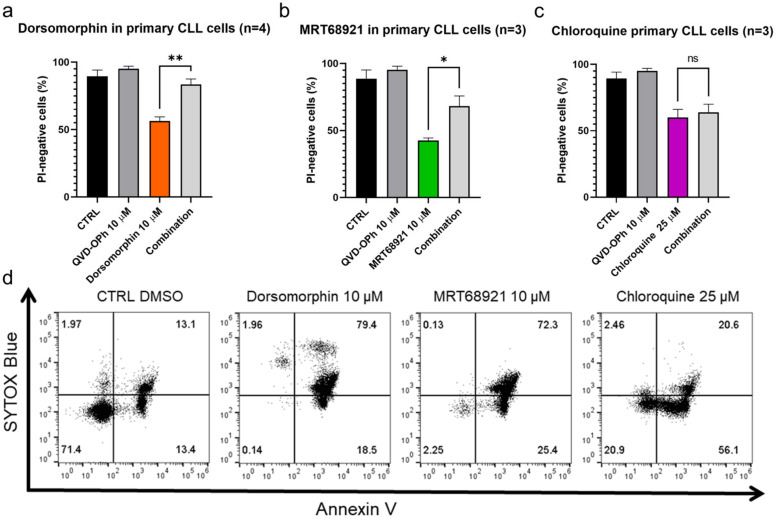
Autophagy inhibitors dorsomorphin, MRT68921, and chloroquine trigger apoptotic cell death in primary CLL cells. CLL cells (1 × 10^6^ cells/mL) were treated with (**a**) dorsomorphin (n = 4), (**b**) MRT68921 (n = 3), and (**c**) chloroquine (n = 3) for 24 h without or with the pan-caspase inhibitor QVD-OPh. Afterward, cells were stained with propidium iodide (PI) and the cell viability was determined by flow cytometry. Data are means ± SEM of ≥3 independent experiments, each carried out in duplicate. Student’s *t*-test was used. Not significant (ns), ∗, and ∗∗ denote *p* > 0.05, *p* < 0.05, and *p* < 0.01, respectively. (**d**) Representative dot plot diagram for phosphatidylserine exposure as a measure of cell death. Patient-derived cells were treated with dorsomorphin, MRT68921, and chloroquine for 24 h, followed by ANV/SB staining.

We show that inhibition of caspases rescued CLL cells from dorsomorphin-induced and MRT68921-induced but not from chloroquine-induced cell death (Figure 3). The viability (propidium iodide negativity) of the control cells and the cells treated with 10 µM QVD-OPh were 89% and 95%, respectively. Treatment of the cells with 10 µM dorsomorphin decreased the cell viability to 56%, while concomitant treatment with QVD-OPh provided significant protection, for the higher cell viability of 84% (*p* = 0.009) (Figure 3a). Similarly, the viability of cells treated with 10 µM MRT68921 was 43% and this was significantly higher (68%) in cells co-treated with QVD-OPh (*p* = 0.0489) (Figure 3b). However, for 25 µM chloroquine, QVD-OPh did not rescue the CLL cells from cell death (Figure 3c).

As these data indicated that the caspase-independent action of chloroquine should be investigated further to better define the mode of cell death, we again treated the patient-derived CLL cells with these three autophagy inhibitors for 24 h and then evaluated the externalization of phosphatidylserine, another hallmark of early apoptosis, using the ANV/SB assay. On the basis of ANV−/SB− indicating viable cells (not undergoing apoptosis), ANV+/SB− indicating early apoptotic or proapoptotic cells, ANV−/SB+ indicating necrotic cells, and ANV+/SB+ indicating dead cells, 13% of the CLL cells were already undergoing measurable apoptosis (ANV+/SB−) and 13% were dead (ANV+/SB+) (Figure 3d). Indeed, this is in line with the fact that CLL cells undergo spontaneous apoptosis when cultured in vitro [25]. Treatment of the CLL cells with 10 µM dorsomorphin, 10 µM MRT68921, or 25 µM chloroquine decreased the proportion of live/nonapoptotic cells from 71% in the vehicle control to 0%, 2%, and 21%, respectively. At the same time, the proportion of proapoptotic cells (ANV+/SB−) increased from 13% in the control to 19%, 25%, and 56%, respectively. This demonstrates that phosphatidylserine is exposed on the outside of the CLL cells following the treatment with each of these autophagy inhibitors, with the greatest effect seen for chloroquine (Figure 3d). As the proportion of ANV−/SB+ cells remained repeatedly low in all treatments (Figure 3d), we excluded necrosis as a primary mechanism of CLL cell death triggered by these autophagy inhibitors.

### 3.3. Autophagy Inhibitors Disrupt Mitochondrial Membrane Potential and Trigger Lysosomal Membrane Permeabilization

**Figure 4:**
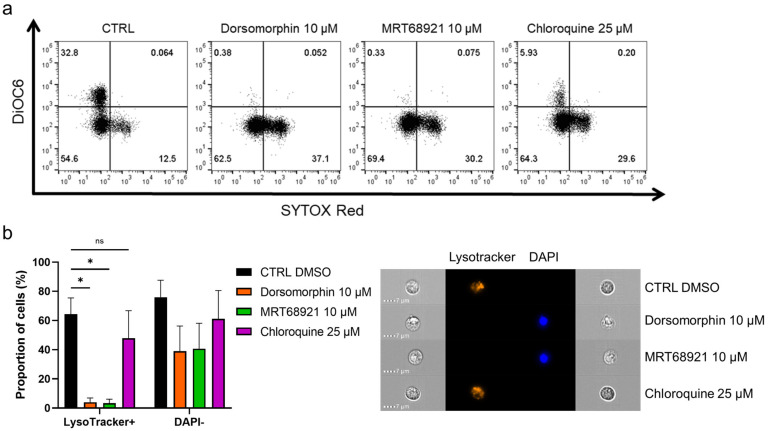
Autophagy inhibitors dorsomorphin, MRT68921, and chloroquine treatments for 24 h disrupt mitochondrial membrane potential and decrease lysosomal integrity in patient-derived CLL cells. (**a**) Representative dot plot diagram of mitochondrial membrane potential and cell viability. (**b**) Autophagy inhibitors perturb lysosomal integrity and decrease cell viability of primary CLL cells. The proportions of LysoTracker+ cells (high lysosomal integrity, viable cells) and DAPI− cells (viable cells) were determined using imaging flow cytometry. Representative cell images are shown. Data are means ± SEM of 3 independent experiments. Not significant (ns) and ∗ denote *p* > 0.05 and *p* < 0.05, respectively.

To further delineate the mode of cell death induced by inhibition of autophagy, we inspected the mitochondrial membrane potential and lysosomal integrity following treatment of the primary CLL cells with the autophagy inhibitors (Figure 4a). Here, the cells were incubated with 10 µM dorsomorphin, 10 µM MRT68921, or 25 µM chloroquine for 24 h, followed by mitochondria staining with 100 nM DiOC6, and cell viability determination with 2 nM SR. The proportion of live cells with high mitochondrial membrane potential (DiOC6+/SR−) was 33% in the control, which is relatively low, but is in agreement with the proapoptotic nature of CLL cells cultured under in-vitro conditions [25]. For the dorsomorphin and MRT68921 treatments, the proportion of DiOC6+/SR− cells dropped to 0%, and for chloroquine to 6%. This was paralleled by increased proportions of live cells with low mitochondrial membrane potential (DiOC6−/SR−) from the control of 55% to the dorsomorphin-, MRT68921-, and chloroquine-treated cells of 63%, 69%, and 64%, respectively. In line with the cytotoxic nature of these autophagy inhibitors, the proportion of dead cells (DiOC6−/SR+) also increased. Thus, treating these CLL cells with the autophagy inhibitors repeatedly and significantly disrupted their mitochondrial membrane potential (Figure 4a), indicating mitochondrial outer membrane potential (MOMP), another hallmark of apoptotic cell death. In short, these data show that MOMP, and therefore apoptosis, occurs in these CLL cells when autophagy is inhibited.

The integrity of lysosomes is critical for cell survival, as permeabilization of lysosomal membranes has been shown to trigger cell death [26]. As lysosomes are an integral part of the autophagic process [27], and autophagic inhibitors can indirectly (dorsomorphin, MRT68921) or directly (chloroquine) affect the physiological role of lysosomes in the autophagic process, we investigated their integrity after exposure of patient-derived CLL cells to these autophagy inhibitors. Following the 24-h treatments, the cells were loaded with LysoTracker and stained with DAPI (Figure 4b). The percentages of LysoTracker+ (viable, high lysosomal integrity) and DAPI− (live) cells seen by imaging flow cytometry demonstrated that 64% of the control cells were positive for LysoTracker. This was greatly reduced by dorsomorphin and MRT68921 (4%, 3%, respectively), with a moderate decrease with chloroquine (48%). These decreases in LysoTracker+ cells were accompanied by decreases in DAPI− cells. The viability of the control cells was 76%, which was reduced by the treatments to 39%, 41%, and 61%, respectively. Thus, it appears that the early autophagy inhibitors dorsomorphin and MRT68921 can significantly impair lysosomal integrity by causing lysosomal membrane permeabilization (LMP); while chloroquine also decreases the percentage of LysoTracker+ cells, but fails to completely perturb lysosomes. This might indicate that targeting autophagy at the earlier stages not only triggers MOMP, but also promotes LMP to trigger apoptosis. Thus, early autophagy inhibitors might show greater antileukemic action in patients with CLL.

### 3.4. Autophagy Inhibitors Are Selectively Cytotoxic toward CLL Cells

**Figure 5:**
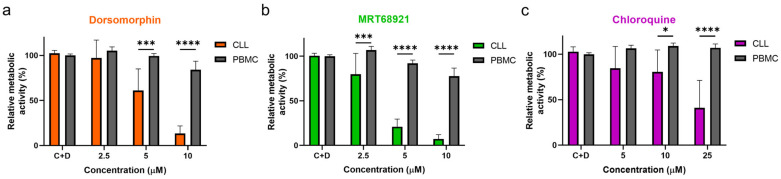
Autophagy inhibitors dorsomorphin, MRT68921, and chloroquine are selectively cytotoxic to CLL cells. CLL cells from 10 patients and PMBCs of five healthy donors (1 × 10^6^ cells/mL) were treated with increasing concentrations of (**a**) dorsomorphin, (**b**) MRT68921, or (**c**) chloroquine for 24 h. Afterward, the metabolic activities of cells were determined using the PrestoBlue assay. Data are means ± SEM of ≥3 independent experiments, each carried out in duplicate. Two-way ANOVA with Sidak’s multiple comparisons test was used. ∗, ∗∗∗, and ∗∗∗∗ denote *p* < 0.05, *p* < 0.001, and *p* < 0.0001 respectively.

To investigate whether the actions of these autophagy inhibitors are directed selectively against malignant cells, the PBMCs of five healthy donors and 10 patients with CLL were treated with increasing concentrations of dorsomorphin, MRT68921, and chloroquine for 24 h. Head-to-head comparisons revealed that these autophagy inhibitors are more potent against CLL cells compared to PBMCs of healthy donors (Figure 5). Dorsomorphin and MRT68921 demonstrated the greatest cytotoxicities against CLL cells, while moderately impacting the metabolic activity of the PBMCs. The metabolic activities of CLL cells treated with 10 µM dorsomorphin, 10 µM MRT68921, or 25 µM chloroquine were 13%, 7%, and 41%, respectively, which were all significantly lower than those for the control PBMCs (84%, 78%, 107%, respectively). Thus, these autophagy inhibitors act selectively against CLL cells, which further substantiates the potential of targeting autophagy for the treatment of patients with CLL.

### 3.5. Venetoclax-Induced Autophagy in CLL Cells Does Not Involve Nuclear Translocation of TFEB

Apart from induction of apoptosis, venetoclax can also interfere with the autophagic pathway by perturbing the Bcl-2/Beclin-1 interaction, which results in the release of Beclin-1 and the activation of autophagy [20]. To monitor autophagic flux following venetoclax exposure, we used the autophagy reporter THP1-Difluo hLC3 cell line, which expresses the RFP::GFP::LC3 fusion protein. These THP1-Difluo hLC3 cells were incubated with increasing concentrations of venetoclax for 24 h and then probed for RFP and GFP fluorescence using imaging flow cytometry. 

**Figure 6:**
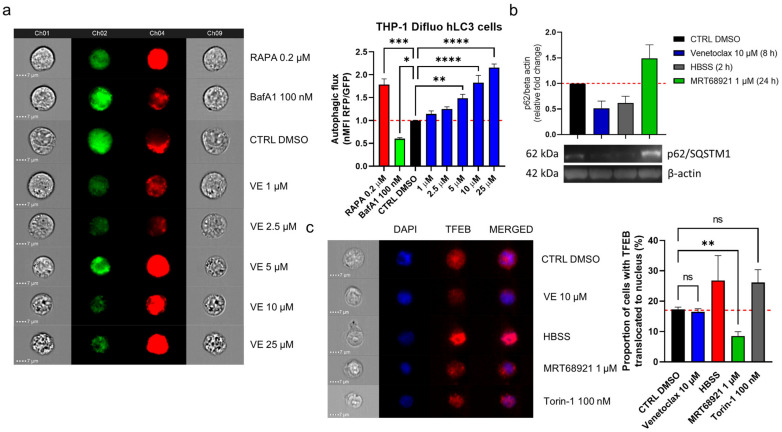
Venetoclax induces autophagy in CLL cells. (**a**) THP1-Difluo hLC3 cells (5 × 10^5^ cells/mL) following the treatments with venetoclax (VE; 1–25 µM), the autophagy inducer rapamycin (RAPA; 200 nM), and the autophagy inhibitor bafilomycin A1 (BafA1; 100 nM) for 24 h. The expression of GFP and RFP were determined by imaging flow cytometry. Data are means ± SEM of 5 independent experiments. Representative cell images for each sample are shown; (**b**) Immunoblot analysis of p62/SQSTM1 in MEC-1 cells (1 × 10^6^ cells/mL) treated with vehicle control (0.2% DMSO) and 1 µM MRT68921 for 24 h, 10 µM venetoclax for 8 h, or HBSS for 2 h. The cells were harvested and lysed, and the proteins were separated by SDS PAGE, blotted onto nitrocellulose membranes, and probed for the expression of the autophagy marker p62/SQSTM1, with the loading control of β-actin. Data are means ± SEM of 3 independent experiments. A representative immunoblot is also shown; (**c**) Representative cell images (left) and quantification (right) of TFEB translocation in MEC-1 cells. The cells (5 × 10^5^ cells/mL) were treated with the vehicle control (0.2% DMSO) or to 10 µM venetoclax, HBSS, 1 µM MRT68921 or 100 nM Torin-1, for 1 h. After, the cells were fixed and permeabilized, and stained with an anti-TFEB antibody and the nuclear stain DAPI. Nuclear translocation of TFEB was monitored using imaging flow cytometry. Data are means ± SEM of ≥3 independent experiments. One-way ANOVA with Dunnett’s multiple comparisons test was used. Not significant (ns), ∗, ∗∗, ∗∗∗, and ∗∗∗∗ denote *p* > 0.05, *p* < 0.05, *p* < 0.01, *p* < 0.001, and *p* < 0.0001 respectively.

Here, venetoclax triggered autophagy in a concentration-dependent manner, starting at a noncytotoxic concentration (1 µM) and at up to 25 µM, where there was morphological evidence of cell death (Figure 6a). To indicate, whether venetoclax induces autophagy in CLL, we treated CLL cell line MEC-1 with 10 µM venetoclax for 8 h (Figure 6b). As the control, the cells were maintained in HBSS for 2 h, to induce starvation and thus autophagy, and treated with 1 µM MRT68921 for 24 h to suppress autophagy. Immunoblotting of these variously treated MEC-1 cells was then performed to determine the levels of the autophagy substrate p62/SQSTM1 (schematically depicted in Figure 1). Here, the levels of p62/SQSTM1 in the venetoclax-treated and HBSS-cultured cells were significantly lower; conversely, there were higher levels of p62/SQSTM1 in the MRT68921-treated cells. The 8-h treatment of the cells with venetoclax induced autophagy as to a similar extent to the 2-hr starvation induced by HBSS, showing that venetoclax can induce autophagy in these MEC-1 CLL cells.

Venetoclax can activate autophagy by perturbing the interaction between Bcl-2 and Beclin-1, which releases Beclin-1 to promote initiation of the autophagic process [20]. However, autophagic flux can also be up-regulated by increased transcription of autophagy-related genes [28]. We thus investigated whether venetoclax promotes nuclear translocation of TFEB, which governs the transcription of crucial autophagy-related genes [28]. To test this, MEC-1 cells were treated with vehicle control (0.2% DMSO), 10 µM venetoclax, HBSS (positive control), 1 µM MRT68921 (negative control), and 100 nM mTOR inhibitor Torin-1 (positive control) for 1 h (Figure 6c). After these treatments, the cells were harvested and probed for the expression of TFEB, and stained with DAPI. Translocation was monitored using imaging flow cytometry.

Here, venetoclax did not interfere with nuclear translocation of TFEB, as the proportion of cells with translocated TFEB remained the same as for the control (Figure 6c). As expected, HBSS and Torin-1 enhanced translocation of TFEB, while MRT68921 efficiently suppressed this.

### 3.6. Perturbing Autophagy Augments the Action of Venetoclax against CLL Cells

Based on these findings that venetoclax can induce autophagy in CLL cells, we postulated that autophagy serves as a mechanism by which the cells resist cytotoxic effects. This would mean that by suppressing the induction of autophagy, the action of venetoclax against CLL cells will be enhanced. To test this, we treated MEC-1 cells with venetoclax, the autophagy inhibitors, and combinations of these for 48 h. The metabolic activities of the cells were then determined using the PrestoBlue assay.

**Figure 7:**
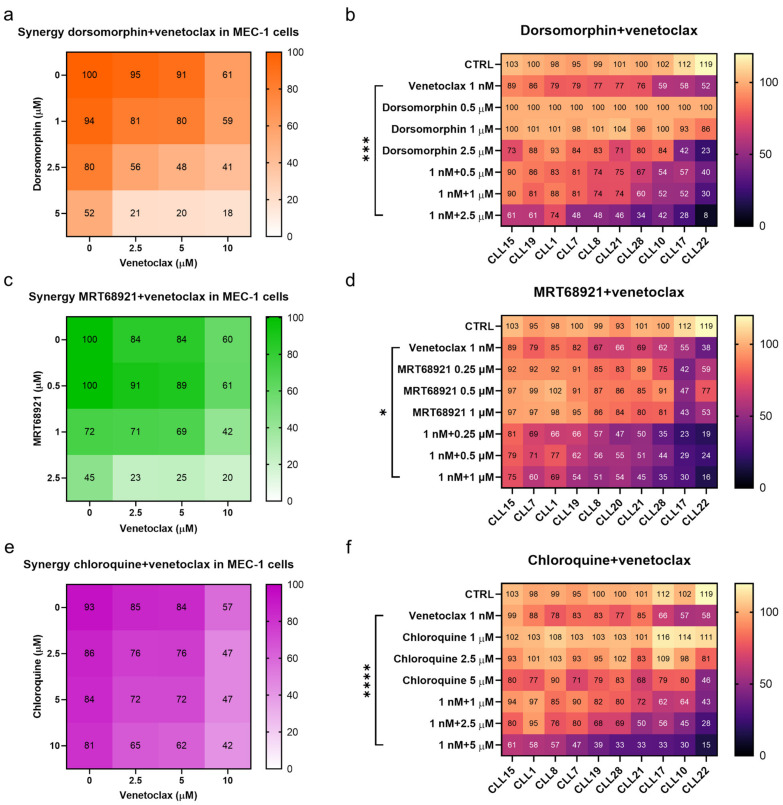
Synergistic effects of autophagy inhibitors and venetoclax. Synergistic effects of dorsomorphin and venetoclax in (**a**) MEC-1 cells and (**b**) primary CLL cells derived from 10 patients; Synergistic effects of MRT68921 and venetoclax in (**c**) MEC-1 cells and (**d**) primary CLL cells derived from 10 patients; Synergistic effects of chloroquine and venetoclax in (**e**) MEC-1 cells and (**f**) primary CLL cells derived from 10 patients. Each square represents the mean relative metabolic activity of cells. For MEC-1 cells experiments were repeated ≥3, each carried out in duplicate. For CLL cells, each column represents responses of individual patient’s cells to different treatments, while each row represents the responses of CLL cells from 10 patients to a specific treatment. One-way ANOVA with Dunnett’s multiple comparisons test was used. *, *** and **** denote *p* < 0.05, *p* < 0.001, and *p* < 0.0001, respectively.

Dorsomorphin and MRT68921 showed the most potent synergistic activities in combination with venetoclax in MEC-1 cells (Figure 7). For example, the metabolic activity of MEC-1 cells treated with 2.5 µM dorsomorphin, 5 µM venetoclax, and their combination were 80%, 91%, and 48%, respectively. This demonstrated that relatively noncytotoxic concentrations of these compounds can synergize to produce potent cytotoxicities against the MEC-1 cells (Figure 7a). Unlike dorsomorphin, MRT68921 acted synergistically with venetoclax at concentrations that were already cytotoxic to cells. The metabolic activities of these MEC-1 cells treated with 2.5 µM MRT68921, 2.5 µM venetoclax, and their combination were 45%, 84%, and 23%, respectively (Figure 7c). Chloroquine also synergized with venetoclax in these MEC-1 cells, however, to a lesser extent than seen for dorsomorphin and MRT68921 (Figure 7e). The metabolic activities of MEC-1 cells treated with 10 µM chloroquine, 5 µM venetoclax, and their combination were 81%, 84%, and 62%, respectively. Overall, all three autophagy inhibitors complemented the action of venetoclax against MEC-1 cells.

As MEC-1 cells cannot represent the diversity that comes with patients with CLL, we also determined the synergistic effects of venetoclax and the autophagy inhibitors on patient-derived CLL cells (from 10 patients). The cells were treated with 1 nM venetoclax, three different concentrations of dorsomorphin, MRT68921 or chloroquine, and also their combinations for 48 h. As for MEC-1 cells, inhibition of autophagy increased the cytotoxicity of venetoclax in these patient-derived CLL cells. However, while synergistic effects were shown for each set of patient-derived CLL cells, the degree of synergistic cytotoxicity varied substantially. The CLL cells that were more prone to venetoclax responded better to the autophagy inhibitors and the synergistic actions of the combination notably reduced the viable fraction of CLL cells (Figure 7). However, even in the patient-derived CLL cells that were only moderately affected by venetoclax or an autophagy inhibitor alone (viability > 80%), the combination treatments managed to kill 40–70% of cells (Figure 7b,d,f), thus suggesting that relatively noncytotoxic concentrations of drugs can have potent cytotoxic actions if they are used in combination.

**Figure 8:**
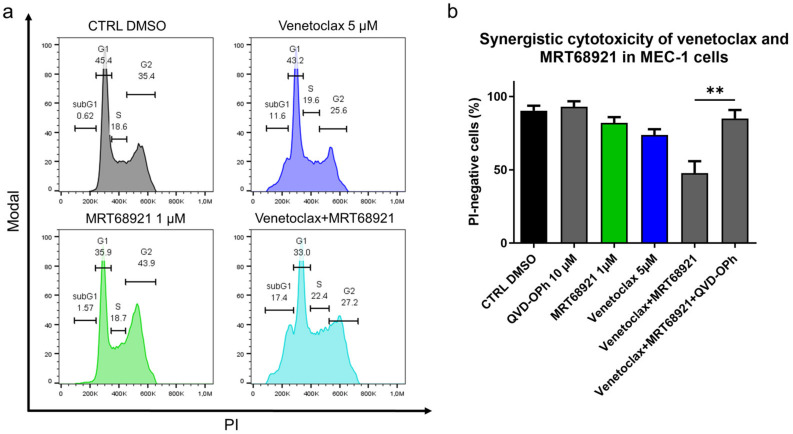
Mechanistic insight into the synergistic cytotoxicity of venetoclax and MRT68921 in CLL cells. (**a**) Cell cycle analysis of MEC-1 cells (3 × 10^5^ cells/mL) treated with 5 µM venetoclax, 1 µM MRT68921, and their combination for 24 h. A representative experiment is shown; (**b**) Caspases are essential for synergistic cytotoxicity of venetoclax and MRT68921. MEC-1 cel cellsls (3 × 10^5^/mL) were treated with a combination of 5 µM venetoclax and 1 µM MRT68921, 10 µM QVD-OPh, and their combination for 24 h. The cell viabilities were determined by 5 µM propidium iodide staining and analysis by flow cytometry. Data are means ± SEM of 3 independent experiments. Student’s *t*-test was used. ∗∗ denotes *p* < 0.01.

As the ULK1/2 kinase inhibitor MRT68921 demonstrated superior antileukemic potential and as it complemented the cytotoxic action of venetoclax most prominently among the tested autophagy inhibitors, we finally investigated the mechanism of its synergistic action with venetoclax. Here, MEC-1 cells were treated with 5 µM venetoclax, 1 µM MRT68921, and their combination for 24 h. Analysis of the cell cycle revealed that venetoclax increased the proportion of cells in the subG1 phase (0.6% vs. 11.6%), MRT68921 increased the proportion of cells in the G2 phase (35.4% vs. 43.9%) (Figure 8a and Figure S2), and their combination shifted the G2 arrested cells into subG1 phase (0.6% vs. 17.4%). This suggested that venetoclax-induced DNA fragmentation (Figure 8a) is preferentially occurring in the MRT68921-triggered G2 arrested cells (Figure S3). As the synergistic cytotoxicity of venetoclax and MRT68921 lies in the triggering of cell death of G2 arrested cells, we postulated that their combined action relies on caspases. MEC-1 cells were thus treated with 5 µM venetoclax and 1 µM MRT68921, 10 µM QVD-OPh, and their combination, for 24 h. The cell viabilities were then determined by using propidium iodide and flow cytometry. This indicated that the synergistic action of venetoclax and MRT68921 required caspases, as QVD-OPh completely blocked the synergistic cytotoxicity of their combination (Figure 8b). This brings to light the central role of apoptosis in the synergistic action of drugs that target Bcl-2 and autophagy.

## 4. Discussion

In the present study, we investigated the therapeutic potential of autophagy inhibitors alone and in combination with venetoclax. To fully grasp the potential of autophagy as a drug target, six distinct autophagy inhibitors were initially screened for their cytotoxic activities against primary CLL cells derived from 5 different patients. By targeting the two metabolic sensors responsible for balancing the autophagic activity in cells, we identified inhibition of AMPK (dorsomorphin), but not activation of mTOR (MHY1485), as sufficiently cytotoxic to these CLL cells. The lack of cytotoxicity of MHY1485 can be attributed to the diverse roles of mTOR, as it governs cell growth, proliferation, and metabolism [29], while at the same time, it negatively regulates autophagy by preventing the interaction of AMPK with ULK1 [30,31]. Despite this limited cytotoxicity, the prosurvival effects that ensue from using mTOR activators limit their use as antileukemic drugs. In this regard, mTOR inhibitors have demonstrated greater potential and are already being investigated for the treatment of various hematologic malignancies, including CLL [32].

The cytotoxicity of the AMPK inhibitor dorsomorphin was surpassed only by the ULK1/2 inhibitor MRT68921, suggesting that the AMPK/ULK1 pathway is particularly important for balancing cell survival and death in CLL. Not only did the inhibition of the AMPK/ULK1 pathway induce MOMP, LMP, phosphatidylserine exposure, and caspase-dependent cell death in these CLL cells, but it was also selectively cytotoxic toward malignant B lymphocytes, while marginally affecting the viability of PBMCs of healthy donors. Moreover, the actions of dorsomorphin and MRT68921 were not affected by the patient clinical characteristics, such as del(11q), del(13q), +12 or del(17p), with MRT68921 demonstrating even better cytotoxicity against cells with mutated IGHV. Collectively, this evidences the potential of targeting the AMPK/ULK1 pathway for the treatment of patients with CLL with diverse genetic backgrounds.

Contrary to AMPK/ULK1 pathway inhibitors, late-stage autophagy inhibitors were less potent in patient-derived cells. Among these, the lysosomal vacuolar H^+^/ATPase inhibitor bafilomycin A1 exhibited no cytotoxicity at concentrations that inhibited autophagy, and demonstrated cytostatic effects in the MEC-1 cells. In contrast, chloroquine had cytotoxic effects against the patient-derived CLL cells. The discrepancies between the two autophagy inhibitors have already been investigated by Mauthe et al., who proposed that cellular effects of chloroquine can, in addition to fusion impairment, also be attributed to disorganized Golgi complex and impaired endo-lysosomal system [33]. On the other hand, the observed antiproliferative effects of bafilomycin A1 can be due to decreased activity of mTORC1, which results from decreased efflux of lysosomal amino acids [34]. As a lysosomotropic agent, chloroquine accumulates in lysosomes and increases the lysosomal pH, which prevents the fusion of lysosomes with autophagosomes, and thus blocks autophagy. Chloroquine was cytotoxic to patient-derived CLL cells at concentrations that inhibited autophagy. We observed MOMP and phosphatidylserine exposure but not involvement of caspases, suggesting that chloroquine triggers caspase-independent cell death. Our findings in patient-derived CLL cells are in line with the findings of Maclean et al. and Zaidi et al., who separately found that chloroquine induces caspase-independent cell death [35,36]. Much like dorsomorphin and MRT68921, chloroquine acted selectively against malignant B-lymphocytes and demonstrated activity against cells of patients independently of their genetic background also against those with poor prognostic markers, such as those with del(11q), del(17p), and unmutated IGHV. This concept brings to light the potential of using autophagy inhibitors for the treatment of hard-to-treat patients with CLL. In the present study, chloroquine was not assessed for its effects on nuclear translocation of TFEB; however, it should be noted that lysosomotropic agents can increase translocation of TFEB; this results in increased lysosomal biogenesis and cancer resistance [37,38], which perhaps indicates that chloroquine is not the best option for treating patients with CLL. In addition to this, the EC_50_ values for chloroquine are approximately 10-fold higher than the observed plasma concentrations (around 1.5 µM) [39], suggesting that chloroquine is not likely to make it as a monotherapy in CLL. However, given the potent synergistic effects observed with 1 and 2.5 µM chloroquine, we propose that chloroquine can be considered for use in combination with targeted therapies, such as venetoclax.

LMP and MOMP have prominent impacts on cell survival, and can occur simultaneously but independently of one another [40]. We demonstrated that both of the upstream autophagy inhibitors dorsomorphin and MRT68921 induced MOMP and LMP in patient-derived cells, while chloroquine evidently induced MOMP, but triggered LMP only marginally. One caveat of this observation might be that dorsomorphin and MRT68921 trigger LMP by acting through a caspase-dependent mechanism. Additionally, as caspases are important for LMP [26], limited LMP following chloroquine treatment of CLL cells is in line with the caspase-independent action of chloroquine. In short, in addition to MOMP, targeting the AMPK/ULK1 pathway can also use LMP to trigger cell death, and can thus produce more stronger antileukemic actions in CLL.

Bcl-2 antagonists are known as direct inducers of apoptosis and in the case of venetoclax, also of autophagy. Venetoclax does this by binding to Bcl-2, which induces the dissociation of the Bcl-2/Beclin-1 complex, which then results in activation of Beclin-1-dependent autophagy [20,21]. We demonstrated that venetoclax-induced autophagy does not affect the nuclear translocation of TFEB, and therefore does not affect the transcription of autophagic genes [28]. We provide evidence that blocking venetoclax-induced autophagy using AMPK/ULK1 pathway inhibitors, or chloroquine enhanced the cytotoxicity of venetoclax in both MEC-1 cells and patient-derived CLL cells. Synergistic effects were seen for all of the patient samples, and for combinations of dorsomorphin and venetoclax; a recent study confirmed their strong synergistic cytotoxicity against CLL cells [14]. However, the present study is the first to demonstrate synergistic cytotoxicity for the combination of MRT68921 and venetoclax, which even surpasses that observed for dorsomorphin and venetoclax. We demonstrated that MRT68921 alone can induce cell cycle arrest in G2 phase, which appeared to be due to delayed chromatin condensation [41], and that the addition of venetoclax to MRT68921 triggers cell death in G2 arrested cells, while the synergistic cytotoxicity of the two depended on the caspases. Overall, this underpins the importance of ULK1 kinase in both cell-cycle progression and CLL cell survival.

The observation that the susceptibility of the CLL cells to venetoclax aligned with the susceptibility of the cells to dorsomorphin, MRT68921, and chloroquine led us to propose that a level of apoptotic resistance can be attributed to increased basal autophagy in CLL cells. To date, only a handful of patients with CLL in Slovenia have been introduced to venetoclax, and so far no one has been identified as resistant. Thus, it remains to be investigated in patient-derived cells whether autophagy inhibitors can restore the susceptibility of CLL to venetoclax.

## 5. Conclusions

In conclusion, we have uncovered autophagy as an important crossroads between cell death and survival with the AMPK/ULK1 pathway being the most promising target for triggering apoptotic cell death in patient-derived CLL cells. The study presented here provides the rationale for considering autophagy as a target, and autophagy inhibitors as potentially new compounds for the treatment of CLL.

## Figures and Tables

**Table 1:** The clinical characteristics of the patients with CLL included in this study.

Patient No.	Gender	Age (Years)	Time from Diagnosis (Years)	Immunophenotype	Mutations					Treatment
					del(13q)	+12	del(11q)	del(17p)	IGHV	
1	M	60	10	CLL	Negative	Negative	Negative	Negative	ND	None
2	M	68	1	CLL	Negative	Negative	Negative	Negative	ND	None
3	F	71	16	CLL	ND	ND	ND	ND	ND	None
4	M	65	11	CLL	Negative	Positive	Negative	Negative	U	None
5	M	58	10	CLL	Positive	Negative	Positive	Negative	ND	Yes
6	M	38	8	CLL	Positive	Negative	Negative	Negative	U	Yes
7	M	54	11	CLL	Positive	Negative	Negative	Negative	U	Yes
8	M	66	0	CLL	Positive	Negative	Negative	Positive	U	Yes
9	M	81	10	CLL	Positive	Negative	Negative	Negative	ND	Yes
10	M	87	1	CLL	ND	ND	ND	ND	ND	None
11	F	80	0	CLL	Positive	Negative	Positive	Negative	U	Yes
12	F	55	2	CLL	Positive	Negative	Negative	Negative	Mut	None
13	M	66	16	CLL	Positive	Negative	Negative	Negative	ND	None
14	M	66	10	CLL	Positive	Positive	Negative	Negative	Mut	Yes
15	F	63	11	CLL	Positive	Negative	Negative	Negative	U	Yes
16	M	79	7	CLL	Positive	Negative	Negative	Negative	Mut	Yes
17	M	73	0	CLL	Negative	Negative	Negative	Positive	U	Yes
18	F	64	5	CLL	Positive	Negative	Negative	Positive	U	Yes
19	M	44	4	CLL	Positive	Negative	Negative	Negative	U	Yes
20	F	72	6	CLL	ND	ND	ND	ND	ND	None
21	M	73	0	CLL	Negative	Positive	Negative	Negative	U	Yes
22	M	79	4	CLL	Negative	Negative	Negative	Negative	ND	Yes
23	M	52	8	CLL	Positive	Negative	Negative	Negative	Mut	None
24	M	63	1	aCLL/MCL	Negative	Negative	Negative	Negative	ND	Yes
25	F	64	4	CLL	Positive	Negative	Positive	Negative	U	None
26	M	75	10	CLL	Positive	Negative	Negative	Negative	Mut	Yes
27	M	71	1	CLL	ND	ND	ND	ND	ND	None
28	F	56	15	CLL	Negative	Negative	Positive	Negative	U	Yes

CLL, chronic lymphocytic leukemia; aCLL/MCL, atypical chronic lymphocytic leukemia/mantle cell lymphoma; IGHV, immunoglobulin heavy-chain variable region gene; M, male; F, female; ND, not determined; U, unmutated; Mut, mutated; del(13q), deletion of long arm of chromosome 13; +12, trisomy of chromosome 12; del(11q), deletion of long arm of chromosome 11; del(17p), deletion of short arm of chromosome 17.

**Table 2:** The EC_50_ values of autophagy inhibitors dorsomorphin, MRT68921, and chloroquine following 24-h and 48-h treatments of the primary CLL cells.

CLL Cells Patient No.	Dorsomorphin (µM)		MRT68921 (µM)		Chloroquine (µM)	
	24 h	48 h	24 h	48 h	24 h	48 h
1	6.4	4.9	3.7	3.4	16.0	5.6
2	5.2	4.4	3.0	2.6	14.0	8.8
3	5.9	3.0	3.7	2.7	23.8	13.7
4	6.5	5.4	4.1	2.3	13.1	10.1
5	7.8	5.5	5.2	4.5	60.1	22.3
6	4.4	3.5	3.2	1.8	60.8	14.3
7	7.3	3.1	4.8	3.4	21.3	7.3
8	6.5	3.7	3.8	2.6	53.1	23.5
9	8.6	5.6	4.8	4.0	19.9	4.1
10	5.0	2.8	4.8	3.3	46.4	7.6
11	3.1	2.0	1.0	0.2	4.1	2.1
12	12.2	11.1	8.0	7.5	46.3	24.0
13	1.8	1.6	1.2	0.7	6.6	4.6
14	14.2	9.6	10.8	9.7	60.4	23.1
15	6.5	4.4	4.5	3.4	29.7	14.3
16	6.4	5.2	4.2	3.4	>100	45.2
17	2.6	2.9	1.6	1.3	28.4	19.4
18	8.5	7.5	5.7	5.2	37.0	24.9
19	12.4	7.6	8.9	6.8	92.7	29.3
20	3.6	3.0	3.6	3.5	21.1	13.4
21	9.0	4.5	5.6	2.5	54.9	16.7
22	2.9	2.0	1.3	1.3	22.4	8.5
23	7.0	5.3	3.7	3.2	32.8	19.1
24	9.9	9.2	6.5	5.0	>100	67.4
25	5.1	3.9	2.4	1.2	51.5	22.0
26	3.5	2.0	4.3	4.0	18.5	9.0
27	3.7	3.1	3.2	3.0	31.9	24.9
28	2.7	2.6	2.4	2.3	30.9	11.9
Mean ± SD	6.4 ± 3.1	4.6 ± 2.4	4.3 ± 2.3	3.4 ± 2.1	34.5 ± 20.8	17.8 ± 13.6

## Data Availability

The data presented in this study are available on request from the corresponding author. The data are not publicly available due to privacy and ethical restrictions.

## Supplementary Materials

The following are available online at https://www.mdpi.com/article/10.3390/cancers13184557/s1, Figure S1: Autophagy inhibitors in the autophagy reporter THP1-Difluo hLC3 cell line, Figure S2: Autophagy inhibitor bafilomycin A1 blocks proliferation of MEC-1 cells, Figure S3: Cell cycle phase distribution in MRT68921-treated MEC-1 cells.
