# Supplementary material for: Targeting Autophagy Triggers Apoptosis and Complements the Action of Venetoclax in Chronic Lymphocytic Leukemia Cells
Source: Cancers (Basel). 2021 Sep 10;13(18):4557. doi: 10.3390/cancers13184557 (PMC8466897; doi:10.3390/cancers13184557)
Supplement: Supplementary file 1 [file cancers-13-04557-s001.zip › Supplementary/Figure S1.pdf]

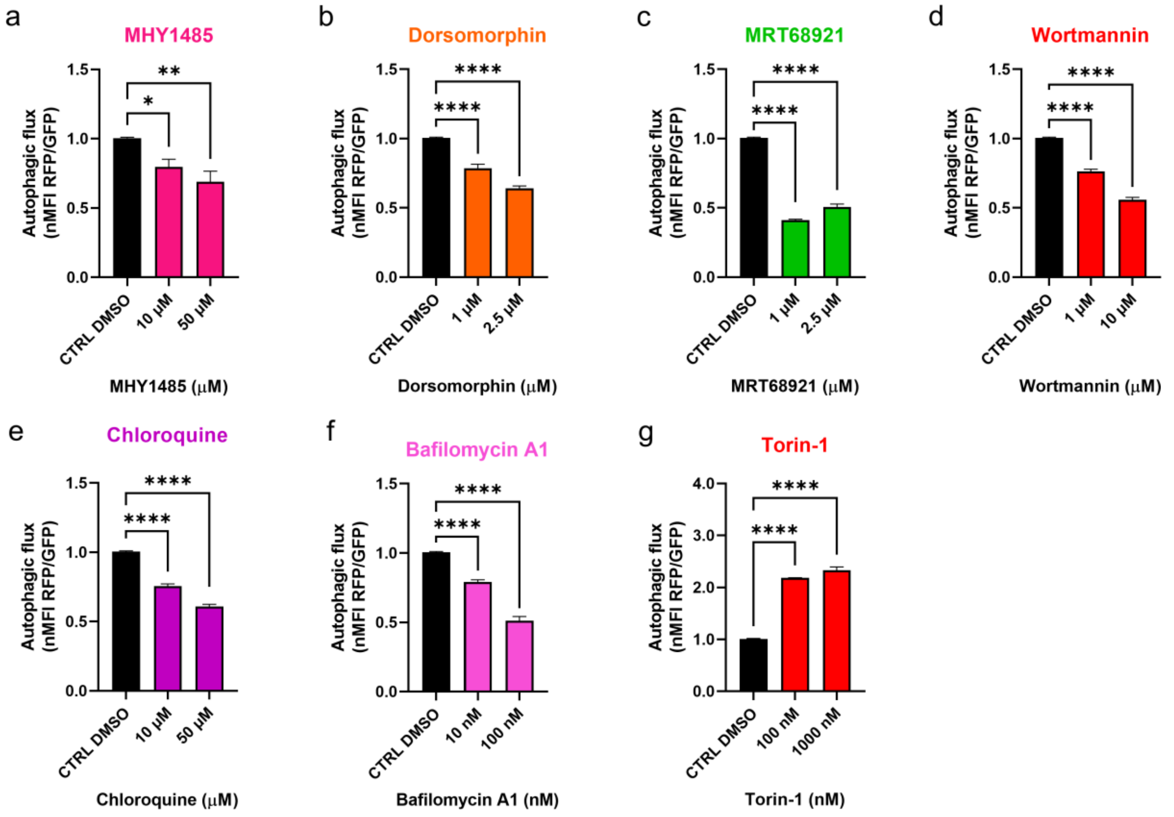

Figure S1. Autophagy inhibitors in the autophagy reporter THP1-Difluo hLC3 cell line. Cells (5×10^5 cells/mL) were incubated with two concentrations of (**a**) mTOR activator MHY1485, (**b**) AMPK inhibitor dorsomorphin, (**c**) ULK1/2 inhibitor MRT68921, (**d**) PI3K class III inhibitor wortmannin, (**e**) autophagosome-lysosome fusion inhibitor chloroquine, (**f**) late-stage autophagy inhibitor bafilomycin A1, and (**g**) mTOR inhibitor Torin-1 for 24 h. Autophagic flux was determined by measuring the fluorescence of RFP and GFP. The ratio of median fluorescence intensity of RFP to GFP (MFI) was normalized (nMFI) to the vehicle control (CTRL DMSO). Data are means ±SEM of 4 independent experiments, each carried out in duplicate.
