# Supplementary material for: Targeting Autophagy Triggers Apoptosis and Complements the Action of Venetoclax in Chronic Lymphocytic Leukemia Cells
Source: Cancers (Basel). 2021 Sep 10;13(18):4557. doi: 10.3390/cancers13184557 (PMC8466897; doi:10.3390/cancers13184557)
Supplement: Supplementary file 1 [file cancers-13-04557-s001.zip › Supplementary/Figure S2.pdf]

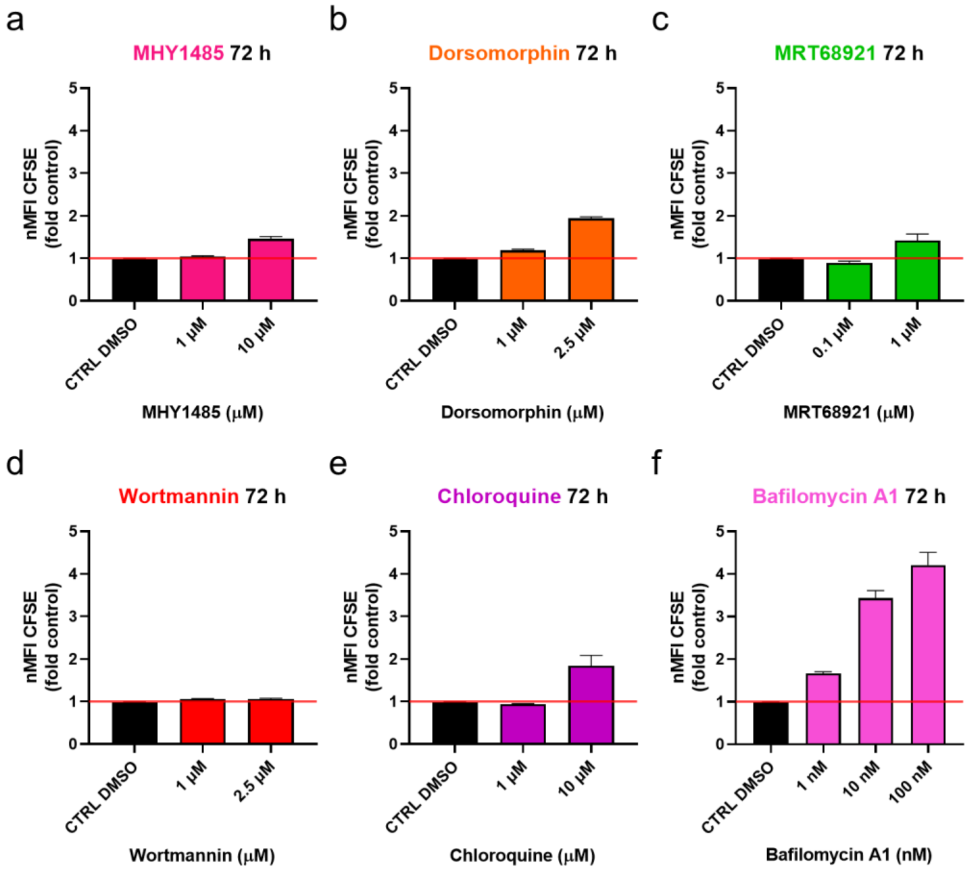

Figure S2. Autophagy inhibitor bafilomycin A1 blocks proliferation of MEC-1 cells. MEC-1 cells (3 ×10^5 cells/mL) were labeled with CFSE and treated with (**a**) MHY1485, (**b**) dorsomorphin, (**c**) MRT68921, (**d**) wortmannin, (**e**) chloroquine, and (**f**) bafilomycin A1 for 72 h. Retention of CFSE was determined using flow cytometry. Data are means ±SEM ≥3 independent experiments.
