# Supplementary material for: Targeting Autophagy Triggers Apoptosis and Complements the Action of Venetoclax in Chronic Lymphocytic Leukemia Cells
Source: Cancers (Basel). 2021 Sep 10;13(18):4557. doi: 10.3390/cancers13184557 (PMC8466897; doi:10.3390/cancers13184557)
Supplement: Supplementary file 1 [file cancers-13-04557-s001.zip › Supplementary/Figure S3.pdf]

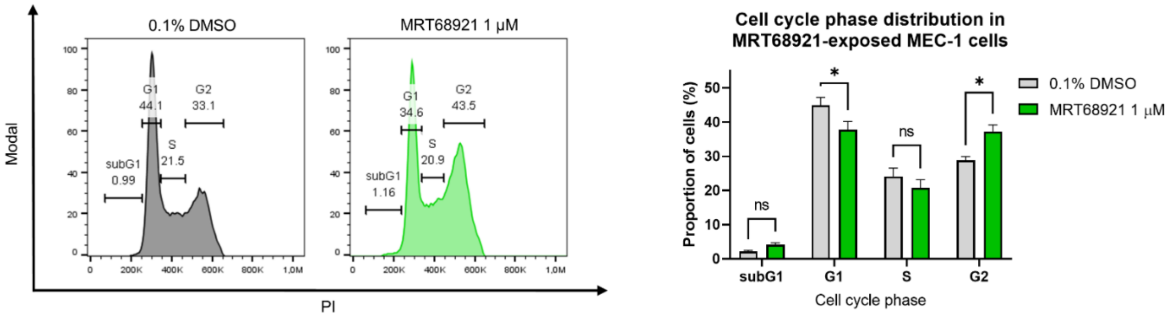

Figure S3. Cell cycle phase distribution in MRT68921-treated MEC-1 cells. MEC-1 cells (3 ×10^5 cells/mL) were treated with the vehicle control (0.1% DMSO) or 1 μM MRT68921 for 24 h. Cell cycle analysis was performed using flow cytometry. Data are means ±SEM of ≥3 independent experiments.
